# Evaluation of Breastfeeding Applications Through the Eyes of Saudi Mothers

**DOI:** 10.7759/cureus.32790

**Published:** 2022-12-21

**Authors:** Thamer M Aledreesi, Ohoud Omar

**Affiliations:** 1 Health Informatics, King Abdulaziz Medical City, Riyadh, SAU; 2 Health Informatics, King Abdullah International Medical Research Center, Riyadh, SAU; 3 Health Informatics, King Saud Bin Abdulaziz University for Health Sciences, Riyadh, SAU

**Keywords:** pediatric nutrition, infant care, motherhood, mobile application, mobile health, health care application, breast milk, m-health, application features, breastfeeding application

## Abstract

Introduction: Breastfeeding is crucial for an infant's health and plays a significant role in mothers' well-being. The recommendation from WHO is to breastfeed your baby for the first six months exclusively, then complementary food is introduced with the continuity of breastfeeding until 24 months or more. Breastfeeding can be challenging, especially for a first-time mom; some mothers also lack knowledge about breastfeeding benefits. Thus, mobile health (mHealth) intervention can raise awareness, provide educational information and emotional support, and offer consultation. Saudi mothers seek an application designed based on their needs.

Objectives: The objective of this study was to analyze the most common breastfeeding application, then extract standard and valuable features. The most useful features were added to the survey and distributed among Saudi mothers. Next, participants' responses to these features were evaluated for recommendation when building a breastfeeding application for Saudi mothers.

Method: This is a quantitative cross-sectional study designed to analyze and explore what Saudi women need in a breastfeeding application to help them make better decisions and provide support. The data was collected through a questionnaire instrument designed after collecting the most valuable features in mobile breastfeeding applications.

Results: The number of Saudi mothers enrolled in this study was 492. Most mothers (90%) were currently breastfeeding or had previous experience with breastfeeding. The participant responses divided results into three categories: essential, recommended, and nice-to-be-added features. One of the highest percentages of important features was adding a section for baby food recipes and how to introduce food.

Conclusion: Saudi mothers need a trustable source of information, consultation, support, and tools to guide them through breastfeeding. With the help of the mHealth application, the breastfeeding experience can be improved.

## Introduction

Breastmilk is the optimum nutrient for infants. It is also an ideal energy source for children between 6-23 months. The World Health Organization (WHO) and the United Nations Children's Fund (UNICEF) recommend exclusive breastfeeding for the first six months of the infant's life. Later, complementary food is introduced while breastfeeding for up to 24 months or more [1].

Breastmilk is clean, safe, and rich in antibodies that help in protecting children from common illnesses [1]. It tends to change according to the baby or child's nutritional and immunological needs. Breastfed babies and children have a lower mortality rate and lower rates of obesity, asthma, sudden infant death syndrome (SIDS), gastrointestinal infections, and severe lower respiratory diseases. Moreover, they score higher on intelligence tests and are linked to higher income later in life [2,3].

Breastfeeding benefits to mothers

Breastfeeding plays an essential role in the well-being and health of mothers. It reduces the risk of ovarian cancer, breast cancer, high blood pressure, and type 2 diabetes [3]. Besides, a study conducted among women in the United States (US) showed that post-partum depression levels were reduced in breastfeeding mothers [4]. Despite all these benefits, only 41% of infants under six months worldwide are exclusively breastfed [5]. Furthermore, the last national survey among Saudi women in 2004-2005 illustrated that only 1.8% of infants are breastfed at 12 months [6].

Factors that affect breastfeeding decisions 

Exclusive breastfeeding seems like the default and ideal method to feed a baby. However, it needs enormous support and could be very challenging. The level of education has no impact on women's decision to breastfeed, but awareness and education about breastfeeding benefits do. Saudi women have limited knowledge of the benefits of exclusive breastfeeding, lack support, and face a negative attitude. There need to be more resources on how to start and aim for successful breastfeeding. This could explain the low rates of breastfed babies in Saudi Arabia. Hence, having consistent, formal, helpful breastfeeding support can enhance exclusive breastfeeding levels and duration [7,8]. The first hour after birth is called a golden hour because the "first milk," or colostrum, is rich in antibiotics. Early education can reflect on early initiation and utilize the most out of the golden hour benefits [9].

Mobile health (mHealth) applications and their role in breastfeeding

Technology and health applications have a positive effect on consumer health. It aims to support consumers and increase the quality-of-care services. These applications facilitate communication between consumers and healthcare providers; also, they allow the consumer to play a significant part in their health. Furthermore, they offer features such as massaging, video, voice calling, and sharing relevant content [10]. Nowadays, consumers use their phones more often, changing how health aids are delivered. Especially in Saudi Arabia, smartphones are widely used among the population in their various daily activities [10]. The availability of advanced communication fifth generation (5G) networks and the popularity of smartphones in the population is an expected enabler for spreading mHealth applications.

mHealth applications can be used to increase awareness and provide support for exclusively breastfeeding mothers. These applications must be introduced in the early trimesters or even before getting pregnant. This will allow mothers and families to make informed decisions and get all the support they need. Additionally, educating pregnant women early would enable them to be emotionally and psychologically prepared [11].

Common mHealth applications for breastfeeding

There are more than 30 applications in Apple Store (Apple Inc., Cupertino, California, United States) and Google Play (Google LLC, Mountain View, California, United States) related to breastfeeding. However, limited breastfeeding applications offer consultation; most applications only provide tracking and reminder tools to help mothers. This section will explore the common breastfeeding mobile applications currently in use.

The LactApp (LactApp Women Health, SL, Barcelona, Spain) is an example of a breastfeeding application that provides consultation through live chat and monitors breastfeeding by tracking an infant's bowel movement and growth. They conducted the most frequent talks in 2019 about breastfeeding techniques, stages, human milk storage and management, breastfeeding myths, infant sleep, returning to work, and mixed feeding. More features are provided by this application, such as blogs related to breastfeeding, infants, and mothers' health. Additionally, there are options for creating plans to achieve different goals like exclusive breastfeeding and returning to work [11].

The Pacify application (Pacify Health, LLC, Washington DC, United States) offers consultation for 24 hours via video and audio visits. The experts are up-to-date and ready to help and educate parents at the touch of a button, with no waiting and no appointment. Different topics and questions can be discussed with them, such as pumping, sore nipples, fever, rashes, gas, teething, sleeping, and crying. Consumers have reported positive feedback and emphasized the 24/7 availability. However, it is not free and requires certain internet speeds and data usage via wifi or at least a third-generation (3G) mobile network to work [12].

The MyMedela Baby Tracker (Medela UK Ltd., Manchester, United Kingdom) supports breastfeeding mothers with an activity tracker, interactive checklist, personalized content, and pumping tips. They offer a Bluetooth connection to their breast pump and track the baby's weight, height, diaper change, and sleep. Sharing data is also possible and consumers can easily share their infant's data with others. The consumer can find educational information and breastfeeding tips on the consumer's page. One of the benefits that consumers pointed out was how the pumping tracker helped them by setting a pumping schedule to boost their milk production [13].

Tools and features that may help to enhance breastfeeding

Researchers have mentioned several features to be effective in breastfeeding applications. Emotional or appraisal support has proven to be beneficial to breastfeeding mothers. Moreover, providing and raising access to an International Board of Lactation Consultant Examiners® (IBLCE®) professional enhanced breastfeeding rates. It could be offered in many ways, such as video or audio calls and live chat. Both personalization and interactivity can enhance engagement and support. Breastfeeding mothers breastfeed exclusively and for longer durations with the help of video consultation and 24/7 availability is critical [14,15].

Offering educational resources such as videos and blogs highlighting the benefits and importance of breastfeeding will help raise awareness, thus reflecting on mothers to make better decisions. In a study conducted on Saudi women in 2018, 46.1% wanted to breastfeed exclusively. Then, they were provided with an educational video emphasizing breastfeeding benefits and early initiation. A positive response was noticed as around 80.8% changed their decision to breastfeed exclusively. So, this educational content was able to alter the mother's choice to better interventions. Finally, about 90% of Saudi women participants revealed that the educational content received from mHealth is valuable in helping the mother to formulate a decision about breastfeeding [7]. 

The content and features Saudi women seek in the breastfeeding application

Determining what content and features Saudi women seek in a breastfeeding application is very crucial. mHealth applications have shown their power in guiding breastfeeding mothers through consultation, educational content, and tracking. It is essential to fully understand what women need to promote breastfeeding to tailor it to their requests. Saudi women might have different perspectives on the features and content they need. Culture, lifestyle, religious beliefs, and other factors may affect the users' preferences. Our aim in this study is to collect and explore the user preferences of Saudi women who used or intended to use breastfeeding application features and recommend the most valuable functions that could be included in any breastfeeding application that serves the women in the kingdom.

## Materials and methods

Most frequent breastfeeding applications were identified in the Apple Store. The applications were chosen based on the high rating and the best download count. All features that improved breastfeeding and guided mothers to decide on breastfeeding exclusively or even for longer durations were collected. A survey was built to discover what features Saudi breastfeeding mothers need in a breastfeeding application.

Study area and subjects

This study is specifically for Saudi women planning to breastfeed or breastfeed mothers. There are no recent percentages on the numbers of Saudi breastfeeding women. However, according to the General Authority for Statistics [16], more than 5 million females are between the ages of 20 and 40. Saudi pregnant mothers intending to breastfeed or those who breastfeed or breastfeed their babies.

Study design and size

This is a quantitative cross-sectional study. A validated questionnaire was distributed among the targeted population to collect the data. The number of Saudi mothers enrolled in this study was 492 participants; 92 mothers had not breastfed earlier and had no intention to breastfeed in the future. Thus, they were excluded.

Data collection, measurement, and analysis

This study was designed to analyze and explore what Saudi women needed in a breastfeeding application to help them make better decisions and support them in breastfeeding. The data were collected through a questionnaire instrument designed after collecting the most valuable features in mobile breastfeeding -applications.

The Research Tool

The questionnaire was a 15-question survey; the first three questions were about demographics. The following questions were about the opinion of the participants about the usefulness of features. The last question asked the participants if they had any other part not mentioned in the survey.

Tool Validation

The survey was reviewed by two informaticians and two certified breastfeeding consultants to test out the accuracy of the statement and the wording clearance. Some rewording and adjustments were made after their feedback. After cleaning and checking the accuracy of the data, the IBM SPSS Statistics for Windows, Version 27.0 (Released 2020; IBM Corp., Armonk, New York, United States) program was used to analyze the data using descriptive statics.

Institutional review board approval

The Institutional Review Board of King Abdullah International Research Center approved the study topic, the data collection method, and the written consent form on August 23, 2021 (approval number: SP21R/375/06).

## Results

This questionnaire was created through Google forms and distributed online among Saudi mothers to explore what they seek in a mobile breastfeeding application. The number of Saudi mothers enrolled in this study was 492; 94 mothers did not breastfeed before and had no intention to breastfeed in the future. Thus they were excluded. In total, 359 (90%) were currently breastfeeding or have breastfed in the past, and 39 (9.8%) intended to breastfeed in the future. The mothers with the highest breastfeeding duration or breastfeeding experience of one to two years were 38.9% and the lowest duration was less than six months with 18.3%. The demographic characteristics of participants and breastfeeding duration are given in Table 1.

**Table 1:** Participants' demographics and previous use of breastfeeding applications

Number of Children	Statistics
0	5 (1.3%)
1-3	314 (78.9%)
3-5	48 (12.1%)
More than 5	31 (7.8%)
Average breastfeeding period	
Less than 3 months	75 (18.8%)
Less than 6 months	73 (18.3%)
From 6 months to 1 year	95 (23.9%)
From 1 year to 2 years	155 (38.9%)
Currently breastfeeding, breastfed before, or intend to breastfeed	
Yes, I am breastfeeding or breastfed before	359 (90%)
Intended to breastfeed	39 (9.8%)
Have you used a breastfeeding mobile application before?	
Yes	44 (11.1%)
No	354 (88.9%)
If you have used one, was it helpful?	
Never used one	219 (55%)
Not helpful	12 1(30.4%)

Overall, 354 (88.9) of participants have never used a breastfeeding application, and only 44 (11.1) used an application before, with 58 (14.6%) thinking it is not helpful. Baby tracker was the most common application mentioned by the participants.

The most common features of breastfeeding applications

The demand for features varied: 304 (76.4) of participants assumed that the live chat feature would be helpful; on the other hand, 10 (2.5%) were neutral, and 84 (21.1%) said no. When asked if they think audio or video consultation would be helpful, most 257 (64.6%) said yes, 30 (7.5) of the participants were neutral, and 111 (27.9) denied it would be helpful, as shown in Table 2.

**Table 2:** Most common features in breastfeeding applications

Features	Yes	Neutral	No
Live Chat	304 (76.4%)	10 (2.5%)	84 (21.1%)
Video Or Audio Consultation	257 (64.6%)	30 (7.5%)	111 (27.9%)
24hr Consultation	325 (81.7%)	13 (3.3%)	60 (15.1%)
Milk Production Tracker	264 (66.3%)	32 (8%)	102 (25.6%)
Baby Sleep Tracker	281 (70.6%)	29 (7.3%)	88 (22.1%)
Baby Weight, Height, Diaper Change, And Feeding Time	281 (70.7%)	44 (11.1%)	73 (18.3%)
Educational Blogs About Breastfeeding Benefits, How To Stay Healthy For Your Baby, The Best Breastfeeding Tips, And How To Increase Your Milk Supply	365 (91.7%)	5 (1.3%)	28 (7%)
Support Group	329 (82.7%)	12 (3%)	57 (14.3%)
Baby Food Recipes And How To Introduce Food To The Baby	367 (92.2%)	9 (2.3%)	22 (5.5%)

Most 325 (81.7%) of the mothers considered that the 24-hour consultation would be effective, further 13 (3.3%) were neutral, and 60 (51.1%) did not see it as effective. The majority of participants, 264 (66.3%), assumed that the milk production tracker would be a helpful tool, 32 (8%) were neutral, and 102 (25.6%) did not think it would be helpful. One of the participants said, "I watch the level of milk production so closely, it started to stress me out." When asked about the baby sleep tracking feature, 281 (70.6%) said yes, 29 (7.3%) were neutral, and 88 (22.1%) said no, it would not be helpful.

The majority, 281 (70.7%) of the mothers, assumed that being able to track their baby's weight, height, diaper change, and feeding time would be efficient, 44 (11.1) were neutral, and 73 (18.3%) disagreed. Moreover, one of the participants had tried this tool before she said, "I thought it would be helpful, but I was too exhausted to write down anything, especially when you are breastfeeding all night."

Furthermore, the participants were asked if they thought educational blogs about breastfeeding benefits, staying healthy for your baby, the best breastfeeding tips, and increasing your milk supply were helpful, the majority 365 (91.7%) agreed, five (1.3%) were not sure, and 28 (7%) said no. Then, they were asked about the support group feature; 329 (82.7) wanted to have this feature, 12 (3%) were neutral, and 57 (14.3) did not like this feature. In addition, 329 (82.7) participants acknowledged that offering baby food recipes and how to introduce food to the baby feature will be helpful, nine (2.3%) were neutral, and 22 (5.5%) disagreed.

Features recommended by participants

The last question was, "Are there any other features you think will be helpful in a breastfeeding application?" Most comments were positive, and only two statements said, "breastfeeding is so easy, no need for an application." Overall, the participants addressed the details they wanted. Most of the features recommended were under the proposed features, such as what food can cause babies' allergies, breastmilk's other benefits, or sleeping techniques for babies. One of the participants mentioned that educational blogs need to be introduced as early as the start of pregnancy. Moreover, participants have shown interest in the application and are looking for a trustworthy source of information consultations in Arabic.

## Discussion

The findings in this study suggest several valued features and provide insights into Saudi mothers' preferred breastfeeding application tools. Those features encourage, educate, and improve the breastfeeding experience among Saudi mothers [9]. We found that most mothers, 155 (38.9%), have breastfed their babies for one to two years; however, in the last national survey conducted in 2004-2005, only 1.8% of infants, a significant reduction from 62% recorded around 30 years ago, were breastfed until 12 months [6]. Considering our sample and the difference in the old published work, we recommend a more comprehensive survey conducted at a national level to provide updated information about breastfeeding in Saudi Arabia.

Essential features

The majority, 92.2%, felt the need to have a feature that offers baby food recipes and tips to introduce food to their baby; such results are aligned with a study published in 2018, which illustrated that mobile applications were beneficial when they provided features such as recipes, meal planning, and many others [17]. Many participants have specified the urgency of this feature in the current study; one mother said: "there is a need to know what type of food I should give my baby, what type of food is suitable in this age, and how can I introduce food."

Many studies have been conducted to prove that providing blogs to educate mothers about breastfeeding benefits has improved their decision-making and enhanced their breastfeeding experience. Moreover, 91.7% of the mothers have shown interest in educational blogs about breastfeeding benefits, how to stay healthy for their baby, the best breastfeeding tips, and how to increase the milk supply; this indeed illustrates the importance of educational content's Impact on mothers' decision to take on breastfeeding, quite similar to a study that demonstrated how 80.8% of mothers have decided to breastfeed exclusively after watching an educational demonstration of breastfeeding benefits [7].

Most of the participants, 82.7%, felt the need to have a support group; it could be a group chat or video call. This finding aligns with the studies that demonstrated emotional support features as useful, practical, and positively impacting mothers [9,14]. Another recommended feature is the 24-hour consultation; several mothers have stressed the importance of consulting a certified breastfeeding consultant, which could be through live chat, audio call, or video call. Several studies demonstrated that mothers breastfed for more extended periods and improved their decision-making abilities due to the previously mentioned features. Further, mothers were more likely to return to work earlier because they had these consultations [9,14]. The features mentioned above are recommended and required by the participants.

Nice-to-have features

Some features are just nice to be added; for example, the baby sleep and milk production tracker are not essential, but some mothers might use them more than others. Thus, adding behavioral sleep intervention tips might be a powerful feature where the mother can reduce sleep deprivation. One article mentioned that this mHealth intervention has positively reflected the sleep patterns of mothers and their infants. Further, leveraging mothers by offering tools for tracking a baby's weight, height, diaper change, and feeding time might produce a more functional application [18].

Features that the participants suggested

Most mothers wanted to add more categories based on their needs and previous experience. One participant proposed adding a section for pregnant women to address their challenges, such as pregnancy progress, nutritious food, support group, and essential exercises. In addition, some mothers stressed on providing a category on breastfeeding latching techniques demonstrated by educational videos and pictures. The last segment could include treating nipple vasospasm and blanching, as some mothers requested. One of the mothers asked for a section on breast pumping that offers information on manual or electric pumps, how to clean the pump, the best techniques, and breastmilk handling. Furthermore, participants suggested adding a section to educate fathers, grandmothers, grandfathers, and anyone in contact with the baby.

Some participants mentioned wanting to see an educational section on how to wean the baby, step by step, and how to provide emotional support for the baby during this time. One excellent suggestion was adding a section called traveling with a baby, which could include tips, what to expect, and how to prepare meals quickly.

A recently published work by Murad et al. explored barriers discouraging Saudi mothers from breastfeeding and mentioned misinformation and lack of medical support among the top obstacles [7]. Our findings in this paper shall help to guide application developers in the kingdom to build applications that provide education and guidance that aid breastfeeding mothers in their time of need and overcome those barriers.

Limitations

This single study cannot be generalized to all Saudi women due to the sample size. However, in this study, we did not aim to generalize the results; instead, we wanted to prioritize the findings to inform the proposed intervention. Another limitation is that only some Saudi mothers have a smartphone and might need to learn how to use an application or have limited access to the Internet, especially Saudi mothers living in rural areas.

Recommendations and future directions

Further studies are needed to assess unmentioned features, such as adding behavioral sleep intervention features. After building the application, we recommend a pilot study to examine the usability and usefulness of the software, as well as to measure the adoption rate. Next, updates can be made based on the findings and the application tailored to fit the current market needs.

## Conclusions

There are a lot of assumed misconceptions regarding breastfeeding. Most Saudi women lack knowledge about breastfeeding benefits, techniques, and processes. Breastmilk is clean, safe, nutritious, and rich in antibodies, thus protecting children from common illnesses. mHealth interventions have proven their efficiency in guiding mothers through their breastfeeding experience. Breastfeeding applications help mothers worldwide make better decisions, such as breastfeeding in the golden hour, exclusive breastfeeding, and breastfeeding for longer durations. Accordingly, there is a need to build a breastfeeding application designed especially for Saudi mothers. Women seek a trustable source of information, consultations, support, and tools to guide them through breastfeeding. This application will have a positive impact on Saudi mothers during breastfeeding time.

## Appendices

**Table 3:** Questionnaire used for data collection

Questions	Options
Do you agree to be a part of this study?	Yes/No
How many children do you have?	0 /1-3/3-5/More than 5
Are you currently breastfeeding\breastfeed before or intending to breastfeed?	Yes/No/Intending to breastfeed (if the answer is no they will not be able to continue the survey)
Have you ever used a mobile application related to breastfeeding? If yes kindly write the name of the application. If you have used a breastfeeding application before, did it help you?	Yes/No, Provide the name, Yes/No/Other
Using a live chat consultation feature in a breastfeeding application will be helpful during your breastfeeding process	Agree/Neutral/Disagree
Using a video or audio consultation feature in a breastfeeding application will be helpful during your breastfeeding process	Agree/Neutral/Disagree
A 24 hours consultation feature in a breastfeeding application will be helpful during your breastfeeding process	Agree/Neutral/Disagree
Using a milk production tracker in a breastfeeding application will be helpful during your breastfeeding process	Agree/Neutral/Disagree
Using a sleep tracker in a breastfeeding application will be helpful during your breastfeeding process	Agree/Neutral/Disagree
Using a weight, height, diaper change, and feeding time tracker in a breastfeeding application will be helpful during your breastfeeding process.	Agree/Neutral/Disagree
Having educational blogs about breastfeeding benefits, how to stay healthy for your baby, the best breastfeeding tips, and how to increase your milk supply will enhance your breastfeeding process	Agree/Neutral/Disagree
Having a support group through an application will be helpful during your breastfeeding experience	Agree/Neutral/Disagree
Providing a section for baby food recipes and how to introduce food to your breastfed baby through a breastfeeding application, will it be helpful?	Agree/Neutral/Disagree
Are there any other features you think will be helpful in a breastfeeding application?	Yes/No, Kindly provide the features
